# Spatio-temporal analysis reveals active control of both task-relevant and task-irrelevant variables

**DOI:** 10.3389/fncom.2013.00155

**Published:** 2013-11-13

**Authors:** Kornelius Rácz, Francisco J. Valero-Cuevas

**Affiliations:** ^1^Department of Biomedical Engineering, and Neuroscience Graduate Program, University of Southern CaliforniaLos Angeles, CA, USA; ^2^Department of Biomedical Engineering, Division of Biokinesiology and Physical Therapy, University of Southern CaliforniaLos Angeles, CA, USA

**Keywords:** motor control theory, redundancy, manipulation, dimensionality reduction, spatiotemporal dynamics

## Abstract

The Uncontrolled Manifold (UCM) hypothesis and Minimal Intervention principle propose that the observed differential variability across task relevant (i.e., task goals) vs. irrelevant (i.e., in the null space of those goals) variables is evidence of a separation of task variables for efficient neural control, ranked by their respective variabilities (sometimes referred to as hierarchy of control). Support for this comes from spatial domain analyses (i.e., structure of) of kinematic, kinetic, and EMG variability. While proponents admit the possibility of *preferential* as opposed to strictly *uncontrolled* variables, such distinctions have only begun to be quantified or considered in the temporal domain when inferring control action. Here we extend the study of task variability during tripod static grasp to the temporal domain by applying diffusion analysis. We show that both task-relevant and task-irrelevant parameters show corrective action at some time scales; and conversely, that task-relevant parameters do not show corrective action at other time scales. That is, the spatial fluctuations of fingertip forces show, as expected, greater ranges of variability in task-irrelevant variables (>98% associated with changes in total grasp force; vs. only <2% in task-relevant changes associated with acceleration of the object). But at some time scales, however, temporal fluctuations of task-irrelevant variables exhibit negative correlations clearly indicative of corrective action (scaling exponents <0.5); and temporal fluctuations of task-relevant variables exhibit neutral and positive correlations clearly indicative of absence of corrective action (scaling exponents ≥0.5). In agreement with recent work in other behavioral contexts, these results propose we revise our understanding of variability vis-á-vis task relevance by considering both spatial and temporal features of all task variables when inferring control action and understanding how the CNS confronts task redundancy. Instead of a dichotomy of presence vs. absence of control, we should speak of a continuum of weaker to stronger—and potentially different—control strategies in specific spatiotemporal domains, indicated here by the magnitude of deviation from the 0.5 scaling exponent. Moreover, these results are counter examples to the UCM hypothesis and the Minimal Intervention principle, and the similar nature of control actions across time scales in both task-relevant and task-irrelevant spaces points to a level of modularity not previously recognized.

## Introduction

Redundancy, and the variability it allows, has traditionally been viewed as the central problem of motor control research (Bernstein, 1967), which can be studied at a variety of levels (e.g., task, muscle, or goal redundancy). Here, we understand the term *task redundancy* to be the availability of infinitely many different *mechanical actions* by the neuromuscular system that can accomplish a given motor task. The totality of these mechanical actions form the goal equivalent manifold, a term coined in John and Cusumano (2007). This differs from *muscle redundancy*, which refers to the multitude of *muscle coordination patterns* producing a same mechanical action (Kutch and Valero-Cuevas, 2011). Multifinger static grasp has been studied extensively because it is a good example of task redundancy (Santello and Soechting, 2000; Latash and Zatsiorsky, 2009; Park et al., 2010; Rácz et al., 2012) since using *n* fingertips to satisfy static force and torque equilibrium of the object grasped is underconstrained (i.e., one can, for instance, squeeze an object harder without translating or rotating it). For multifinger grasp, the redundant task space of all applicable forces for static grasp can be mathematically separated into the mutually orthogonal subspaces of force variability that have no effect on static equilibrium (e.g., squeezing the object in static grasp) on the one hand, and on the other hand, force variability that disrupts static equilibrium (i.e., violates the task constraints). Others and we refer to the former and latter subspaces as task-irrelevant (or null space) and task-relevant, respectively, as they indicate a distinction about where the controller is thought to place emphasis.

Proponents of the Uncontrolled Manifold (UCM) and Principle of Minimal Intervention hypotheses have suggested that, to simplify the control task, the nervous system only needs to identify and control the task-relevant subspace, and can disregard the task-irrelevant subspace (Scholz and Schoener, 1999; Scholz et al., 2002; Jordan, 2003; Valero-Cuevas et al., 2009; Latash et al., 2010). Compelling evidence for this comes from spatial domain analyses showing clear structure in the spatial variability of task variables. By *spatial variability* we mean the amplitude and range of the multidimensional task variables of fingertip or resultant forces. Researchers, including our group, have repeatedly shown that the spatial variability in task-irrelevant dimensions is relatively larger than in task-relevant dimensions (Scholz and Schoener, 1999) in analyses of kinematic (Tseng and Scholz, 2005), kinetic (Santello and Soechting, 2000), and EMG variability (Valero-Cuevas et al., 2009). In this context, larger spatial variability in a task dimension is assumed to imply less control effort (i.e., intervention) of those task variables that do not affect the successful performance of the task. In practice, however, even task-relevant dimensions will exhibit some variability because a certain amount is acceptable given, say, high contact friction, or unavoidable, given, say, sensory or motor noise, or neural delays. Conversely, task-irrelevant dimensions will also show some control action when, for instance, noise, delays or stochasticity drive the system across some boundary that requires intervention (e.g., Insperger, 2006; Milton et al., 2009b). Therefore, the relative magnitude of variability across task variables is not necessarily a robust predictor of task-relevance, control action or strategy (Valero-Cuevas et al., 2009; Dingwell et al., 2010). In fact, even proponents of the UCM hypothesis admit the possibility of *preferential* as opposed to a strict separation into clearly controlled and uncontrolled variables (Latash et al., 2010). Despite this qualification, we lack specific quantification and description of controlled intervention in both task-relevant and task-irrelevant spaces that would allow us to understand neural control strategies better.

### Spatial versus temporal variability

There is a growing emphasis to infer neural control strategies by supplementing spatial quantification of variance with temporal analyses. As described above, much more attention has been given to spatial variability. However, relatively little attention has been directed at the temporal structure of variability in task variables in the context of task redundancy (Valero-Cuevas et al., 2009; Dingwell et al., 2010; van Beers et al., 2013). By *temporal variability* we mean the time-varying features of the multidimensional task variables, e.g., fingertip or resultant forces in this case. Lest the reader think that time-varying actions during static force production or grasp is an oxymoron, others and we have shown that finger muscles and fingertip forces exhibit rich dynamics during static grasp (Santello and Soechting, 2000; Valero-Cuevas et al., 2009; Rácz et al., 2012). Being considered and called uncontrolled, the implicit and explicit assumption is that task-irrelevant variability exhibits the spatial and temporal properties of uncontrolled dynamical processes. In the anomalous diffusion literature, this is considered either a white noise process, consisting of uncorrelated samples, or Brownian motion, formed by the integration of the former (Ben-Avraham and Havlin, 2000; Kantz and Schreiber, 2004). In the context of neural control, we take it to mean the state of least control (i.e., truly uncontrolled where the dynamics of the plant is not influenced by the controller). Conversely, a controlled process, continuously or intermittently (Collins and De Luca, 1994; Guckenheimer, 1995; Milton et al., 2009a; Suzuki et al., 2012), will exhibit the temporal properties of controlled dynamical processes such as negative correlations between time samples (i.e., if a task variable moves in one direction, at some future time it will require a corrective action in the opposite direction). Please also note that the mechanical properties of the musculoskeletal plant act as filters on the neural input, and can give to correlations in the output. This is a limitation common to all studies of neural commands. Therefore, studying the force variability that naturally occurs in static grasp provides unique opportunities to reveal the time-varying nature of control actions without having the confounding, or at least superimposed, effects of additional dynamics coming from other features of more dynamical tasks such as gait (Dingwell et al., 2010). By applying a combination of temporal and spatial analysis techniques to multifinger static grasp, we find that task-relevant and task-irrelevant variables are both subject to strong and weak control actions at different time scales. Therefore, these results provide evidence against the UCM hypothesis and the Minimal Intervention principle. We conclude that it is necessary to revisit and revise our understanding of variability vis-á-vis task relevance when inferring control action and understanding how the CNS confronts task redundancy.

## Methods

We combine linear spatial approaches and non-linear temporal approaches to (1) quantify the spatio-temporal nature of the variability in both the task-relevant and task-irrelevant subspaces; (2) compare them to the mechanical predictions of necessary control actions for the task; and (3) evaluate them in light of the UCM hypothesis and Minimal Intervention principle. We selected the task of static tripod grasp because it is a common and useful redundant motor task, and a fundamental aspect of human manipulation (Yoshikawa and Nagai, 1991; Flanagan et al., 1999; Rácz et al., 2012).

### Data collection

**Figure 1:**
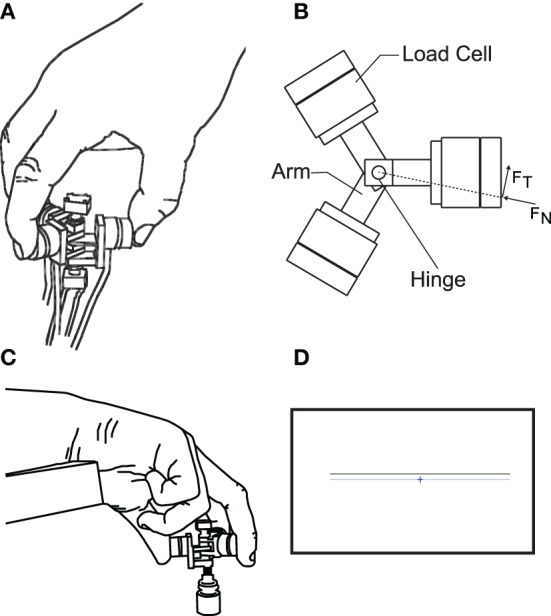
**The apparatus for tripod grasp designed and built in our lab**. It consists of three arms rotating about a common hinge to adjust to the most comfortable configuration for each subject. The arms are then fixed to create a rigid object. Each arm is instrumented with a 6-axis force transducer that forms the contact surfaces for tripod grasp. **(A)** Representative illustration of the instrumented device being held. **(B)** View from above onto the instrumented device, showing the three load cells mounted on arms that rotate about a common hinge, and the forces used in the analysis, computed from the load cell surface measured forces. **(C)** The holding posture during the trials, also showing a weight attached from below to the instrumented device. **(D)** Visual feedback presented to the subjects: a crosshair representing the instantaneous sum of normal forces, to be aligned with a target line.

We asked 12 young, healthy and consenting subjects (ages 20–36, 6 males, 9 right-handed) to perform a static tripod grasp of an instrumented rigid object designed and built in our lab (Figure 1), whose use has been reported in Rácz et al., (2012). While performing the grasp, the thumb, index and middle finger were in contact with three ATI Nano17 6-axis force transducers (Apex, NC, USA) locked in a configuration comfortable for each subject. The angle between index and middle finger was approximately 30°, while the angles formed with the thumb by each finger were approximately 165°. Each force transducer was coated with a Teflon surface to reduce reliance on friction by the subjects to achieve a stable grasp. The force transducers were connected to a 16-bit National Instruments 6225 M-series data acquisition card (National Instruments, Austin, TX, USA). Attached to the object were three markers for motion capture, forming an equilateral triangle, whose plane was parallel to the grasp plane of the three fingertips. Seven motion capture cameras (Vicon, Oxford, UK) allowed us to measure the object's position and orientation to quantify how well the subject met the task goal of maintaining a simple static grasp.

Furthermore, three different weights (50, 100, and 200 g) were attached from below to the object (Figure 1C). Additionally, the latter half the trials were performed with visual feedback presented to the subjects approximately 1 m away on a 23 inch computer screen. The visual feedback consisted of a horizontal target line representing the target sum of normal forces (in Newtons) applied by three fingers, and a crosshair representing the actually applied sum of normal forces (Figure 1D). The goal in those trials with visual feedback was to align the horizontal component of the crosshair with the target line and keep the variability of force application minimal. The target force was the average sum of normal forces applied by subjects across all trials without visual feedback. In effect, the visual feedback added another task-relevant dimension to the task, besides keeping the grasp as static as possible.

Subjects performed all trials with their dominant hand determined as per Oldfield (1971), as shown in Rácz et al. (2012). Subjects were seated in a chair, with the grasping hand resting on the chair's armrest (Figure 1). Moreover, we asked subjects to immobilize the wrist of their grasping hand by gripping the wrist with their non-dominant hand to minimize wrist rotation and hand translation, since we were interested in the coordination of fingertip forces for steady-state static grasp with as little motion as possible.

Subjects performed three repetitions of static grasp trials of 68 s duration for each weight and each visual condition, for a total of 18 trials per subject (3 × 3 × 2). The instructions to the subjects were to simply hold the object in a static tripod grasp with as little motion as possible, as in Figure 1. Even though the object was light (max. 260 g), we provided subjects with 1 min of rest to avoid fatigue or discomfort. Trials were block randomized: the different weights were attached in random order for each condition, but the nine trials with visual feedback were always performed after the ones without. This was because the target total grasp force line height was based on the self-selected average sum of normal forces for each weight in the non-visual condition. The individual experimental conditions are described in Table 1.

**Table 1:** **Overview of the experimental conditions (number of trials in parentheses)**.

**Presented on screen (50 Hz, 1.5 m away)**	**Weight attached to object from below**
No visual feedback (9)	50 g (3)
	100 g (3)
	200 g (3)
Force target tracked by crosshair (9)	50 g (3)
	100 g (3)
	200 g (3)

The instructions to the subjects were to simply hold the object in a static tripod grasp with as little motion as possible, as in Figure 1

### Data preprocessing

The three-dimensional force data recorded by each transducer were sampled at 400 Hz, while the motion-capture marker positions were sampled at 200 Hz (both force and motion data collection were triggered synchronously). We removed the first seven and last 1 second(s) from each trial's time series to avoid transients. Next, we downsampled both the force and motion capture time series to 100 Hz, to balance the need for temporal resolution and computational cost. Having performed the same analysis on a subset of the trial at the original sample rate, we subsequently found that results were unaffected, when repeating the analysis at lower sample rates. Hence, 100 Hz was found to be a useful compromise as it still allows for a physiologically meaningful temporal resolution on the order of 10^−1^ s.

As is required by our temporal analysis, see below, we did not filter the data to avoid creating artifactual correlations.

### Data analysis—spatial

**Figure 2:**
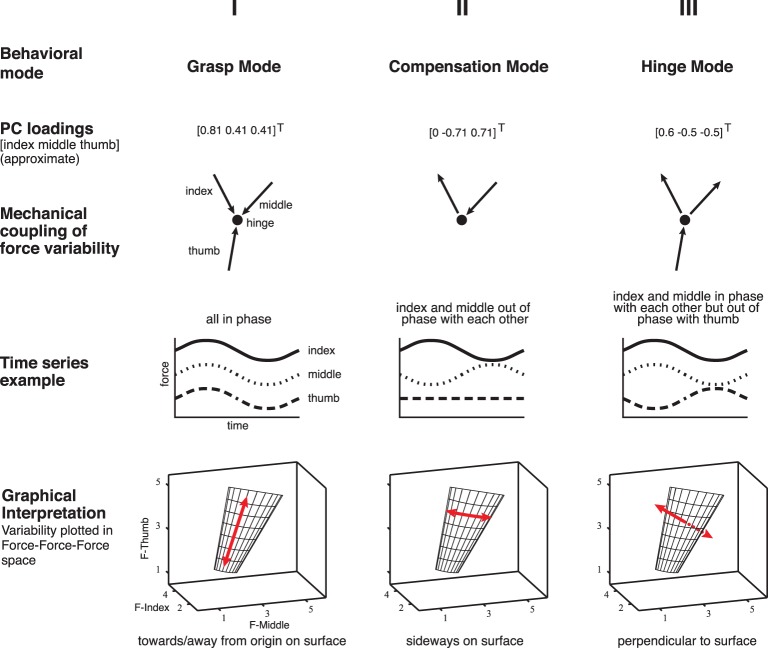
**Illustration of the three Modes of normal forces associated with the principal components computed from the data and the simulations, across all subjects, and conditions [adapted Rácz et al., (2012)]**. Please note that the loadings are the unit vectors describing the multidimensional correlation defining each PC. Therefore the loadings for this PC show that the thumb, index and middle finger forces all co-vary in this Mode. We refer to these three PCs as: (i) the task-irrelevant *Grasp Mode*, along [0.81 0.41 0.41]^*T*^, as it reflects synchronous increases and decreases in the three normal forces, which are also known as grasp forces, (ii) the *Compensation Mode*, along [0.0 −0.71 0.71]^*T*^, reflecting the out-of-phase opposition, or compensation, of thumb normal force by either the index or middle finger normal force, and (iii) the task-relevant *Hinge Mode*, along [0.6 −0.5 −0.5]^*T*^, reflecting an increase (decrease) in thumb normal force accompanied by a simultaneous decrease (increase) in the index and middle finger normal forces, which would typically occur if the object was accelerated by the thumb, thus violating the mechanical task requirements of static grasp (without loss of generality, the violation of static grasp by purely rotating the object using tangential forces is not considered here, see Discussion).

To analyze the spatial coordinated action among the three fingertip forces, we first performed principal component analysis (PCA) on the time series of each sensor's normal forces for each trial. PCA is a popular linear method for the estimation of spatial correlation structures in data (Clewley et al., 2008). Specifically, we computed the three principal components (PCs) of the 3 × 3 normal force covariance matrix (q-PCA). Each PC is a unit vector whose elements, called loadings, specify the multidimensional correlation among variables; and a combination of PCs forms a basis defining a vector subspace that is a linear approximation to the spatial correlation structure in the data (Clewley et al., 2008). PCA has been commonly used to estimate effective degrees of freedom in motor systems, and in the context of the UCM hypothesis to compute task-relevant and -irrelevant latent variable spaces, which are represented by the orthogonal PC vectors (e.g., Santello and Soechting, 2000). We then projected the 3-dimensional normal forces (one normal force per force sensor) time series data onto the three principal components. Following Rácz et al. (2012), we call the first, second, and third principal components the Grasp, Compensation and Hinge Modes of this task (Figure 2), respectively. We also tested doing this same analysis on the full 3D force data (normal and two tangential force components per force sensor, see Discussion and Figures 10–13) but the results are unchanged from when using only the normal force component from each sensor, in particular since the magnitude of the tangential force fluctuations were several orders of magnitude smaller than those of the normal forces, but not their mean levels, since vertical tangential components are required to sustain the weight of the object against gravity. Importantly, adding tangential forces to the analysis adds several task-relevant or task-irrelevant dimensions, which however, does not affect the fundamental question or findings of this study, i.e., the implications of certain temporal dynamics for the study of control of task-relevant and -irrelevant dimensions.

### Data analysis—temporal

Next, we applied Detrended Fluctuation Analysis (DFA) to each projected time series (Kantelhardt et al., 2001) to detect *temporal correlations in non-stationary time series*. It has the advantage, in particular over the classical time-lagged autocorrelation function, that it can distinguish unwanted trends of arbitrary order that can give rise to spurious non-zero correlations, from actual long-range correlations in non-stationary data. Examples of non-stationary data are time-series with trends that are long relative to the length of the time series or which exhibit clustering—mathematically speaking, data whose two-point autocorrelation is time-variant. DFA has been used extensively for the analysis of behavioral and physiological data (Hausdorff et al., 1996; Peng et al., 1998; Penzel et al., 2003). Mathematically, it quantifies the power-law increase of the root-mean square deviations from a trend in the time series fluctuations, once segments of increasing length *n* have been subtracted from it to remove trends of that length:

$$F(n)={\left[\frac{1}{L}\sum_{j\;=\;1}^{L}{\left(X_{j}-(a_{j}+b)\right)}^{2}\right]}^{\frac{1}{2}}$$

Where *X*_*j*_ − (*a*_*j*_ + *b*) represents the residuals of the linear fit *a*_*j*_ + *b* to the time series segments *X*_*j*_ of length *n*. For a given segment length *n*, there are *L* overlapping segments in the process. The complete expression for *F*(*n*) represents the average root mean square deviation at segment length, or time scale, *n*. In a non-stationary process, this time scale is related to *F*(*n*) by the relationship

$$F(n)\propto n^{\alpha }$$

This power-law increase in root-mean square deviation is mathematically linked to long-range temporal correlations in the data: negative correlations will, over time, lead to a smaller rate of increase than positive correlations. The scaling exponent α indicates the type of correlation, as well as the strength of the relationship between data increments separated by a time scale *n*. DFA reveals empirically (i.e., in a model-free way with minimal assumptions) the inherent time scales for which different temporal correlations exist in the data by showing if the scaling exponent α [i.e., the slope of the logarithmic plots of *n* vs. *F*(*n*)] differs at different time scales. These time scales are found based on regions of slope linearity in the logarithmic plots of *n* vs. *F*(*n*), and thus regions of actual power-law scaling.

In particular, the scaling exponents α can be fit to the logarithmic plots of the time scales *n* vs. the *F*(*n*) (for an interpretation of these scaling exponents, see Table 2).

**Table 2:** **Different scaling exponents found by linear fitting in the logarithmic displacement vs. time scale plot**.

**Value of** α **at *n***	**Type of correlation**	**Nature of correlation**	**Effect on data**
>0.5	Persistence	Positive (negative) increment followed by positive (negative) increment	Expansion
<0.5	Anti-persistence	Positive (negative) increment followed by negative (positive) increment	Contraction (stabilization)
=0.5	Brownian motion	No correlation between increments	(No control)

Because long-range negative correlations reflect corrective actions that prevent dissipation, they are interpreted as evidence for the workings of corrective and stabilizing (i.e., negative feedback) control, while positive correlations can be interpreted as evidence of lack of corrections and thus lack of stabilizing control actions (Collins and Luca, 1993; Collins and De Luca, 1994). (Please note that these notions are related, but not equivalent to notions of stability, which are beyond the scope of this work because our static grasp task is stable). Recent work also supports the idea of interpreting scaling exponents in terms of indicating the degree of control effort (Dingwell and Cusumano, 2010; Dingwell et al., 2010).

To further confirm the reliability of our results, we repeated the DFA on the first and second half of each trial to test if the structure of the variability in normal forces is sensitive to the level of total grasp force. We felt this to be necessary because, as is commonly reported in studies of static grasp (e.g., Johansson and Westling, 1987), we noticed that some trials exhibited a relaxation of the total grasp force, likely an adaptation to reduce fingertip forces over time to mitigate fatigue (see Results).

### Modeling of tripod grasp

As in Rácz et al. (2012), we applied the same analysis methods to synthetic data generated by a simulation of the task. For a description of the model, see Appendix. In that model the variability in the simulated normal forces comes from our implementation of a standard Brownian random walk [see Appendix and Rácz et al. (2012) for details]. Analyzing data from a strictly mechanical simulation allows us to disambiguate features of mechanical origin from features of the control that cannot be explained by mechanics, and are therefore of likely neural origin [for other examples of this approach see Kutch and Valero-Cuevas (2012), Rácz et al. (2012), and Ristroph et al. (2010)].

## Results

### Principal component analysis of simulated normal forces

**Figure 3:**
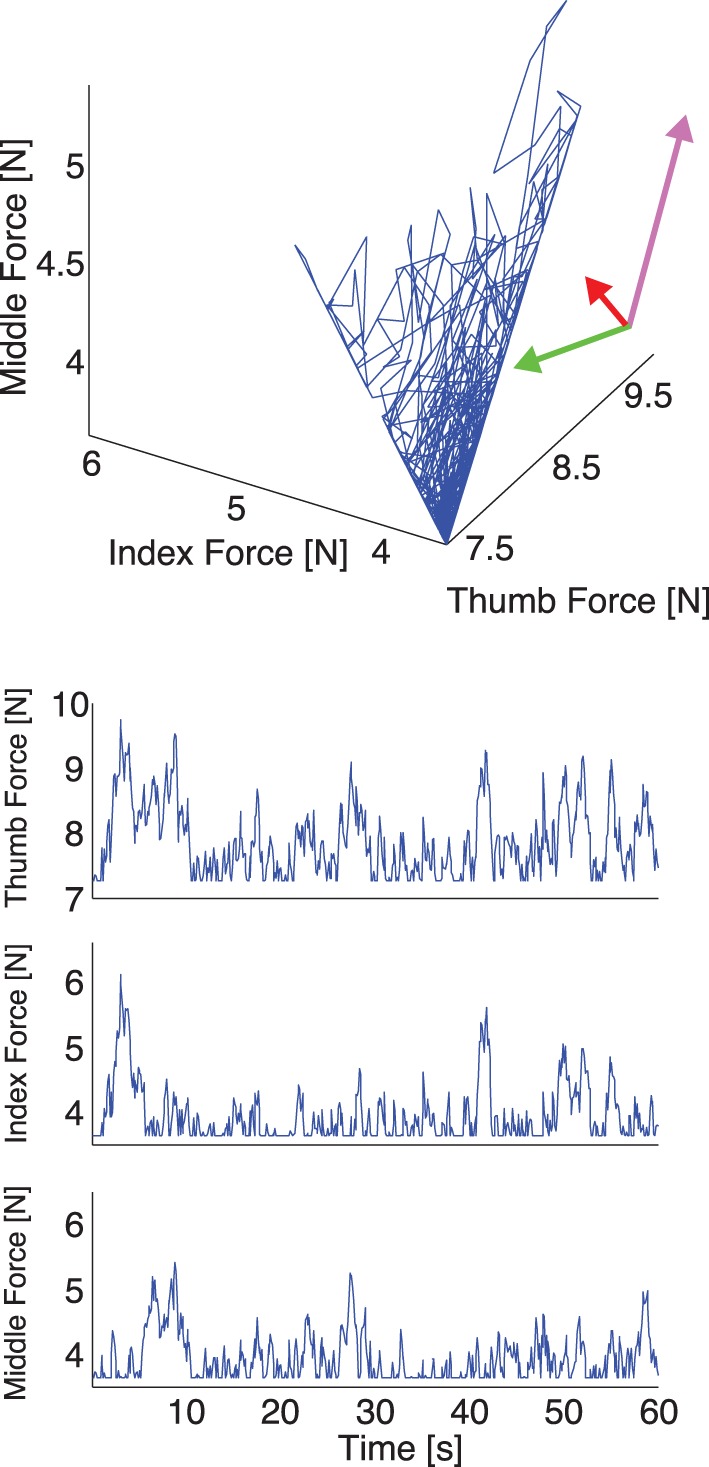
**Representative plot of the simulated thumb, index, and middle finger normal forces *without* visual feedback. Top:** The three simulated normal forces plotted against each other. Note that the force fluctuations come to lie on a plane, whose orientation we compute using PCA. The rotated coordinate system indicates the directions of normal force variability, and the lengths of the arrows indicate the variance explained along that direction. Note that since in the simulation the motor task is executed perfectly, two directions, which span a manifold of solutions, explain all variability. **Bottom:** The three simulated normal forces during a trial plotted individually. Note that the floor effect results from the hard constraint of minimum normal force in the simulation, which for the subjects is more flexible and can result in a downward trend in total grasp force in trials without visual feedback.

Figure 3 shows the simulated normal forces plotted against each other, which shows that, by construction, the valid solutions populate a plane representing the constraints of the task. In agreement with our mechanical analysis (Rácz et al., 2012), PCA of the simulated data finds the two basis vectors (principal components, or PCs) describing that plane: the Grasp Mode [0.81 0.41 0.41]^*T*^ and the Compensation Mode [0.0 −0.71 0.71]^*T*^, Figure 2.

Mechanically, the dynamics associated with the Compensation Mode reflects movement of the intersection point of the three force vectors, as shown in Yoshikawa and Nagai (1991) and Flanagan et al. (1999): as long as the force vectors, extended from their respective application points, intersect in one common point inside the object, there will be no moment exerted on the object. The only physical limitation is that the force vector extended from each fingertip stay within its friction cone. The Grasp Mode, in turn, quantifies changes in the total grasp force, which is equivalent to the intersection point not moving side-to-side on the manifold in Figure 2, but rather up-and-down as the distance to the origin quantifies the total grasp force.

These two PCs together explain all the normal force variance in the simulated data. In this idealized case, by construction once again, if the variability of normal fingertip forces exhibits this structure in steady-state static tripod grasp, then such variability will not give rise to accelerations or rotations of the grasped object and exists entirely in the null space of the task. Actual acceleration of the object is associated with variability of normal forces perpendicular to this plane, along the PC vector of the Hinge Mode [0.6 −0.5 −0.5]^*T*^.

### Principal component analysis of experimental forces

As expected, subjects met the task requirements of not dropping the object and holding it still, but still showing some variability in their normal forces and object movement. The object markers (for motion capture) stayed well within 5 mm in all directions, and object motion was significantly affected by the presence of visual feedback, but not weight (*p* < 0.01, Mann–Whitney U test). Given the mechanics of the task and instructions to the subjects, the small but measurable linear accelerations of the object must be due to dynamics along the Hinge Mode (or to a lesser extent to the unmodeled vertical motion and 3D rotation modes given that the wrist was held fixed).

**Figure 4:**
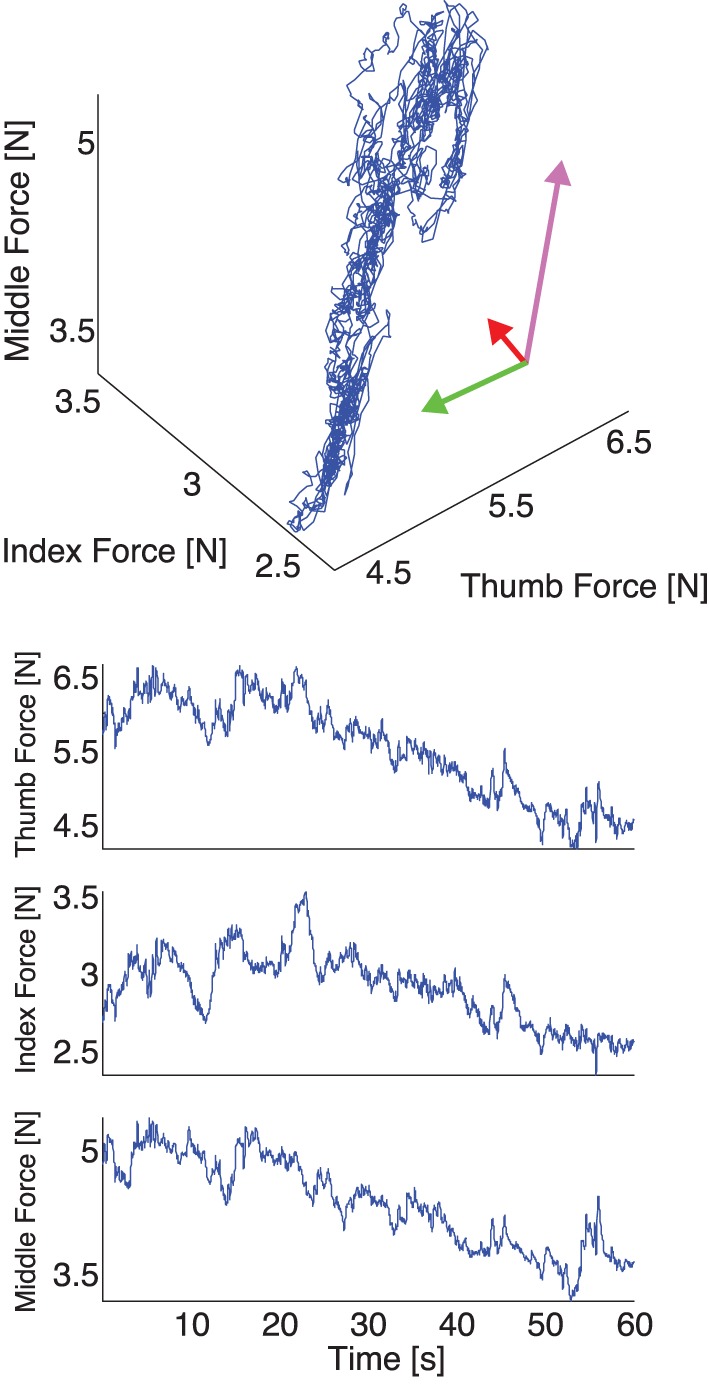
**Representative plot of experimental thumb, index, and middle finger normal forces recorded during one trial with a 200 g weight *without* visual feedback. Top:** The three normal forces plotted against each other. Note that the force fluctuations come to lie on a plane defining the mechanical requirements of the task (see Appendix and Figure 2), whose orientation we calculate using PCA (directions and variances explained shown by the rotated and scaled coordinate system—note that most of the variability is explained by two components and the data come to lie on a plane). Note the elongated distribution of the data, due to a gradual reduction of total grasp force in the absence of visual feedback. **Bottom:** The three normal forces during a trial plotted individually. Note the elongated distribution of the data is here seen as a downward trend in the three fingers.

**Figure 5:**
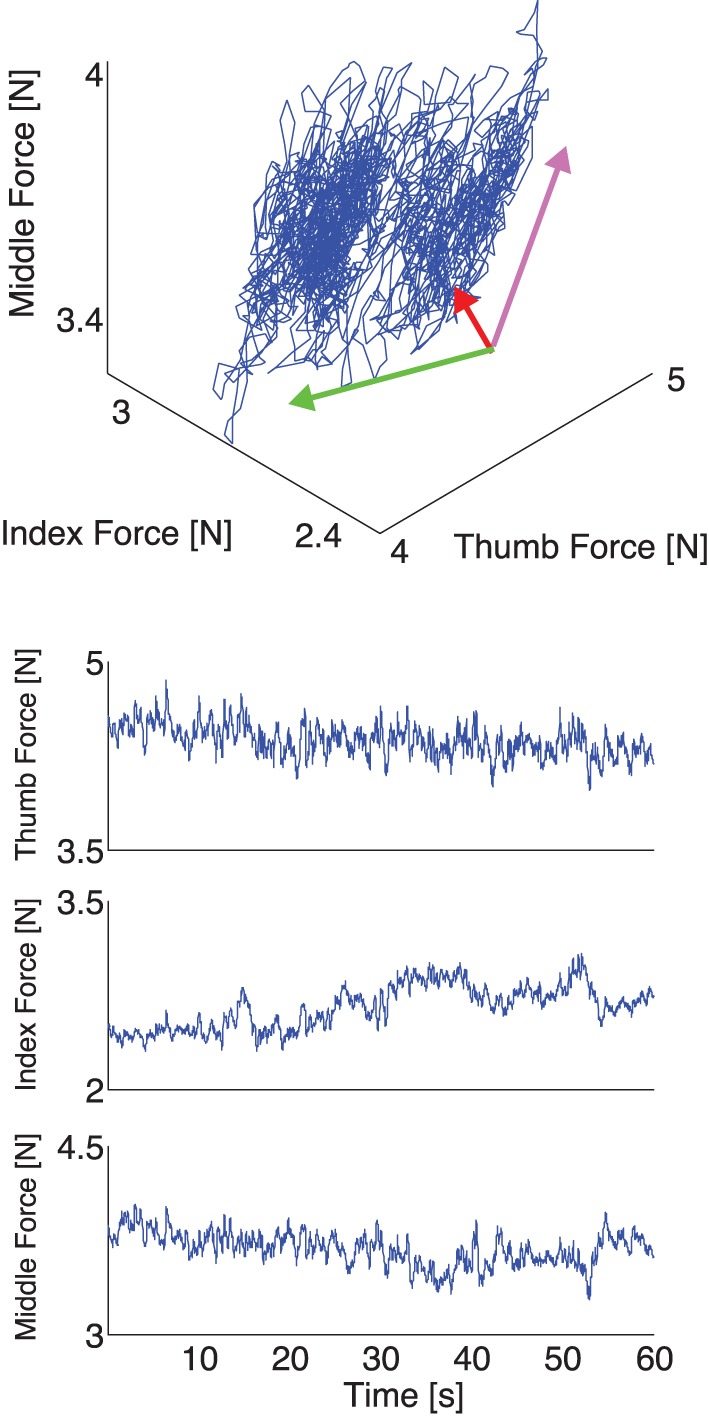
**Representative plot of experimental thumb, index, and middle finger normal forces recorded during one trial with a 200 g weight, *with* visual feedback. Top:** The three normal forces plotted against each other. Note that the force fluctuations come to lie on a plane, as is expected if the task constraints are met (see Appendix and Figure 2, note the rotated and scaled coordinate system), but the variability in normal forces populates the plane in a tighter cluster given that a constant total grasp force is now a task constraint the subjects enforces. **Bottom:** The three normal forces during a trial plotted individually. Note the absence of a downward trend across the three fingers, due to the enforcement of the visual constraint on the sum of normal forces.

We applied PCA to the time series of experimental normal forces (see Figures 4, 5 for representative trials for two different conditions) and, as expected from the mechanical requirements of the task (Rácz et al., (2012), we found that the variability of normal forces consistently exhibited a structure described by the three principal components found in the simulation.

**Figure 6:**
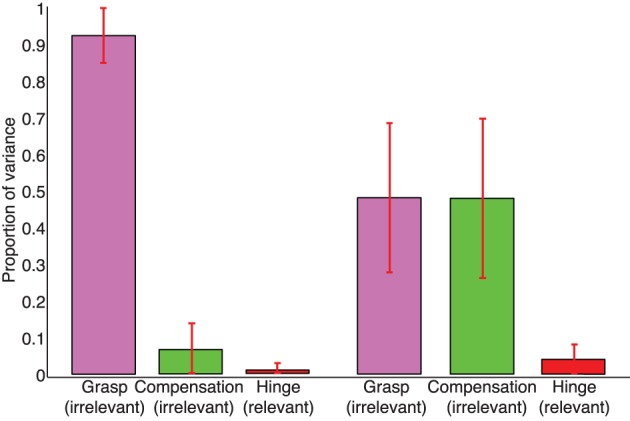
**Mean proportions of variance explained by the Grasp, Compensation, and Hinge Modes, respectively, in the sample trials *with* (*right*) and *without* (*left*) visual feedback**. In trials without visual feedback, PCA indicates that most variance occurs along the Grasp Mode—which is true given that subjects gradually reduce the total grasp force and the data are distributed in an elongates fashion compared to the tighter cluster in the case where visual feedback is provided (cf. Figures 2, 3) The overwhelming majority of the variance in trials with and without visual feedback is explained by the Grasp and Compensation modes, and hence the normal force variability occurs on a planar manifold.

In the case of no visual feedback, the Grasp Mode obtained from PCA explains approximately 90% of the normal force variance, while the Compensation Mode approximately 5–10% and the Hinge Mode 1–3% (Figure 6). In contrast, in trials with visual feedback the Grasp and Compensation Modes contribute roughly equally to the normal force variance, slightly less than 50% each (Figure 6) with 1–3% accounted for by the Hinge Mode. The low percentage of variance explained by the Hinge Mode in both cases shows that subjects were mindful of the request to perform static grasp, and satisfied the task requirements of not accelerating the object. Lastly and not surprisingly, the Hinge Mode shows almost no variation over time given that the object was held relatively still, as confirmed by motion capture. Figure 10 further shows that the variance explained by three Modes remains unaffected even if we consider all three force components for each digit.

### Detrended fluctuation analysis of time series projected onto principal components

Our first finding is that the Grasp, Compensation and Hinge Modes all naturally exhibit three distinct scaling regions, representing temporal correlations at three different time scales (Figure 8). In particular, the distinct time scales are at 10, 100, and 1000 s of milliseconds, subject to some fluctuation. Due to this fluctuation, we calculated the scaling exponent only for a conservative subrange of these time scales that was common to all trials and subjects, i.e., 1–50, 250–500, and 3500–7000 ms.

In the following, all reported changes in scaling exponents α (i.e., slopes of the log–log plots) are statistically significant at the *p* < 0.01 level, based on Kruskal–Wallis (across the three weight conditions) and Mann–Whitney U statistical tests (across the two visual feedback conditions). We used these non-parametric test (equivalents of ANOVA and *t*-test, respectively), because inspection of deviations from normality revealed a clear absence of a normal distribution of α required for parametric tests.

### Detrended fluctuation analysis: general scaling exponent results

Consider Figure 9, which shows the mean scaling exponents across all trials, respectively. At short time scales (1–50 ms), the slopes associated with both the Compensation and Hinge Mode time series are close to 0.5, indicating lack of positive or negative correlation (approximating a random walk) between increments and thus absence of a corrective control effort, while the Grasp Mode has a mean slope of 0.7, reflective of positive correlations (i.e., diffusive growth) in the time series.

At medium time scales (200–500 ms), the slope of the Grasp Mode decreases to 0.5, indicating lack of corrective control effort along this dimension, while the Compensation Mode now indicates the activity of a stabilizing or correcting effort, with the scaling exponents α having decreased to a value of 0.3, and the Hinge Mode shows a very strong negative correlation (indicative of strong corrective action) of RMS deviation scaling with exponent α = 0.1, indicating a strong tendency to enforce a constant mean level. Importantly, the 200–500 ms time delays include the shortest voluntary time scales of the sensorimotor system (Kawato, 1999).

The long time scale (3500–7000 ms) is not particularly different from the 200–500 ms time scale in terms of DFA slopes, except that the Grasp Mode now becomes corrective as well, with a slope having decreased from 0.5 to 0.3.

Importantly, DFA scaling exponents did not significantly differ between the first and the second half of the trials.

### Effect of adding visual feedback

Solid arrows in Figure 9 show the effect of adding visual feedback. Note that these arrows indicate only those statistically significant changes found based on our Mann–Whitney U statistical tests. Visual feedback had the predictable effect of decreasing the scaling exponent α for the Grasp Mode at the long time scales of 3500–7000 ms; indicating the success of the long visuomotor loop in keeping the total grasp constant. However, and somewhat counter-intuitively, it also increased the slope of the Grasp Mode at short time scales (1–50 ms), indicating greater positive correlations (i.e., diffusive growth) in the short latencies not affected by the visuomotor loop. This may reflect increased signal-dependent noise and spurious corrections known to result from higher gains in the motor and sensory components of a feedback loop—in this case the visuomotor loop. The Hinge Mode was the only other Mode affected by visual feedback; where its slope in the long time scales became slightly, but statistically significantly, more corrective as it is changing from 0.13 to 0.1.

### Detrended fluctuation analysis: effect of increasing weight

Dashed arrows in Figure 9 show the effect of adding weight to the object. Note that these arrows indicate only those statistically significant changes found based on our Kruskal–Wallis statistical tests. The α slope of the Grasp Mode at scales (1–50 ms) increased toward to 1.0, as in the case of adding visual feedback. Again, this perhaps reflects the increase in signal-dependent noise with the need for greater grasp forces. Signal-dependent noise scales linearly with force and is observed in the 8–12 Hz frequency band of force measurements (Jones et al. (2002), i.e., time scales of <125 ms) and induces positive mechanical correlations across fingers due to reaction forces. The only other significant effect of weight was a slight increase of the Hinge Mode slope in the medium time scales, possibly reflecting the increased difficulty of maintaining immobile the more massive objects, which would show less effective corrections in this time-scale (see Figure 9).

## Discussion

Our spatio-temporal analysis of static grasp demonstrates that fingertip forces exhibit evidence of corrective actions and absence of corrective actions in both the task-relevant and task-irrelevant task subspaces. Our main message is that, during a static tripod grasp, we find examples at different time scales of how task-irrelevant parameters, which are commonly associated with the UCM, are actively controlled, and how task-relevant parameters (i.e., performance variables) are not actively controlled. This evidence critically extends our approach to task relevance, and compels us to revise our understanding of neural control of task redundancy. In particular, our results challenge the currently dominant approaches to redundancy of the UCM Hypothesis and the Minimal Intervention principle that advocate a separation of control strategies between task-relevant and task-irrelevant variables. Rather, we demonstrate that there exist corrective actions common to all task variables that supports the notion of a continuum, rather than a separation, of neural control strategies common to both task-relevant vs. task-irrelevant variables. Moreover, the similarity of control actions across time scales seen in both task-relevant and task-irrelevant spaces points to a level of modularity in corrective action not previously recognized. After explaining how methodological considerations do not challenge our main findings, we discuss the implications of our results to our understanding of neural control of task redundancy.

### Methodological considerations

We find that variability of the normal forces of the fingertips on the object during static grasp suffices to show a counter example to current thinking about neural control of task redundancy. We designed our experimental paradigm of static equilibrium to sidestep methodological and theoretical difficulties encountered by prior studies of more complex tasks, e.g., (Dingwell et al., 2008, 2010; van Beers et al., 2013). Studies investigating the UCM hypothesis and Minimal Intervention principle must restrict themselves to a measurable subset of performance variables (it is not practical to record EMG from all muscles, angles from all joints, etc.) during well-defined tasks (like planar limb motion or body motion in the sagittal plane). We used a mechanical model developed in Rácz et al. (2012) to interpret our normal force data, and were careful to only analyze trials for which the linear and angular accelerations were measured as negligible based on motion capture data, and thus considered as static grasp. We initially analyzed the 9-dimensional system that included tangential forces of all three fingertips, but found that the only significant tangential forces were those counteracting gravity. They were relatively constant, which is not surprising given the trials we considered as valid examples of static grasp. The magnitudes of the fluctuations of the other tangential forces (those in the horizontal plane) were several orders of magnitude smaller than the normal forces, and therefore considered negligible for the purposes of making our main point. Namely, showing a counter example of task-irrelevant task variables (those associated with the UCM) being actively controlled during a static tripod grasp. Tangential forces add to the dimensionality of the motor control task—some of the performance variables associated with these dimensions could be identified as task-relevant, others as being part of the UCM. However, any additional task variability dimension is, mathematically and in the context of the UCM hypothesis, perpendicular to existing dimensions (e.g., moment cancelation efforts do not necessitate normal force variability, from a purely mechanical point of view). Therefore, whether or not these additional dimensions are subject to control (i.e., constitute additional performance variables) has no bearing on our main finding that there exist at least two task-irrelevant (from a UCM point of view) dimensions of variability (i.e., Grasp and Compensation Modes) that are being continuously controlled in the task of static tripod grasp, while simultaneously, there exists a task-relevant direction, or performance variable (i.e., Hinge Mode), that is not controlled at short time scales. Nevertheless, studying potential coupling between mechanically independent task dimensions is a worthwhile problem. In fact, we have looked at this problem for a similar (but dynamic) task in a previous paper (Rácz et al., (2012), but that analysis and discussion is beyond the scope of this work.

Our methodology has some important strengths and differences compared to prior work that uses a temporal analysis. Our work on multifinger manipulation differs from that of locomotion (Dingwell et al., 2008, 2010), reaching and gaze shifting (van Beers et al., 2013) in that: (1) it is substantially simpler problem than locomotion and therefore easier to identify performance variables; (2) it is equally important to activities of daily living; and (3) particularly relevant to human evolution. In particular, Dingwell et al. (2010) recently showed that gait, a non-linear dynamical task, exhibits the expected greater variability along goal-irrelevant directions as per the UCM and Minimal Intervention principle. In agreement with our findings, they find corrections for deviations in both goal-relevant and -irrelevant directions; but prefer to say that the nervous system largely “ignores non-essential variations.” While they use DFA to study the correlation structure along each projected time series, they interpret the scaling exponents as continuous variables that indicate different levels of control action at different time scales in different subspaces. Given the complexity and non-linearity of their task, they explore variations in model structure to alter what was being controlled, but not the task variables, to further strengthen their conclusions. We did not need to do that because we chose a simpler task where the analytical solution to the mechanics of the system and task allows us to define our Modes, and interpret the scaling exponents. Importantly, they cite us (Valero-Cuevas et al., 2009)—when stating that quantification of variances along spatial dimensions alone can lead to incorrect conclusions about control—as motivation for their use of temporal analyses as a necessary next step. This is the point we also now make by emphasizing spatio-temporal analyses for static grasp. In fact, it is perhaps a testament to the utility of these spatio-temporal analyses that, even when done at multiple levels of observation and across multiple tasks, different studies agree that temporal dynamics is critical to proper interpretation of neural control. Lastly, van Beers et al. (2013) study two simultaneous discrete movement tasks: reaching and gaze shifts between visual targets that are not related to our work in multifinger grasp. However, their autocorrelation analysis of task-relevant and task-irrelevant variables shows that task-irrelevant variability is corrected less intensively. Because their tasks are dynamical target-driven tasks, their interpretation of the temporal structure of variability in the task-irrelevant variables is motor exploration, learning and performance optimization. Given that our static grasp task is simpler and has clear goals that can be modeled mechanically, we can make stronger claims as to the nature and structure of the variability. Our approach is, however, necessarily silent about methodological issues in those other non-linear dynamical experimental and analytical paradigms. However, we agree with them in that active exploration for fatigue mitigation is a potential benefit of variability in these task-irrelevant variables (see below).

Furthermore, it is important to consider prior studies that have identified voluntary and involuntary collaborative force interactions among fingertips when pressing or grasping rigid objects [e.g., Baud-Bovy and Soechting (2001); Scholz et al. (2002); Shim et al. (2005); Latash and Zatsiorsky (2009); see for review Schieber and Santello (2004)]. From the mechanical perspective, many extrinsic flexor and extensor muscles are multitendoned or have multiple compartments subject to a certain level of common neural inputs [but the thumb and index finger are largely independent (Brand and Hollister, 1999)]. This provides a level of mechanical coupling across fingers—which is mostly known to prevent large, individuated or disparate finger motions [Agee et al. (1991); Brand and Hollister (1999); Zilber and Oberlin (2004); as reviewed by Schieber and Santello (2004)]. Our task was designed to consider these potential confounds by requiring a low-magnitude static grasp in postures where all fingers are similarly flexed so that tendinous interconnections do not play a dominant role. Common neural inputs to muscles across fingers are also not a confound because, as reported by Latash and Zatsiorsky (2009), those common drives do not produce the kind of variability that leads to a pervasive dynamic Grasp Mode in the low frequency range during non-grasp force production tasks. Common neural input, by definition, is composed of highly correlated short-latency (i.e., high frequency) discharge of motor units. As reported by Bremner et al. (1991) the duration of the synchronization ranged from 5 to 31 ms (mode = 13 ms). These latencies are only applicable to the shortest (i.e., 1–50 ms) time scales in Figure 9. Moreover, the Grasp Mode captures such effects of common neural drive because it is defined as synchronous increase or decrease of finger forces. Common neural drive would not enter the other Modes because they require opposing (i.e., synchronous increases and decreases) in finger forces. Thus, common neural drive cannot explain our findings of evidence of control action in task-irrelevant variables, and lack of it in task-relevant variables, that are spread across Modes and time scales.

Lastly, Bryce and Sprague (2012) have urged caution when analyzing non-linear or non-stationary signals with DFA. However, our goal is not to estimate exact or specific Hurst exponents, but rather show that a clear deviation from the 0.5 line exists, much like in the recent work by Dingwell. We did consider the potential confound of non-stationary time series, but our results are robust with respect to analyzing first and second halves of each trial. Furthermore, we do not observe an initial curvature mentioned by Bryce and colleagues, among other things because we do not allow for estimation of very small time scales, as mentioned in our methods. Instead, we see an initial linear region, with scaling different for each Mode. This finding is robust across subjects and trial halves. This underscores the stability of our conclusion: that task-irrelevant dimensions are indeed subject to control intervention, and vice versa, and that this observation is time-invariant.

### Spatial analysis

Our simulation results clearly show that the first two principal components, the Grasp and Compensation Modes, span the null space of force dynamics associated with successful static grasp: variation of force inside this manifold does not violate the constraints of static grasp (i.e., zero net force and moment). Given however, that noise and variability are inevitable elements of neuromuscular systems, successful task completion naturally leads to the population of the null space manifold, and task-relevant variability in the Hinge Mode orthogonal to the solution manifold (i.e., modulating linear motion of the object in violation of the static task requirement) will be minimal, but not necessarily zero.

In the case of static grasp, the fingertips are coplanar in the horizontal plane, and their vertical tangential components serve to cancel gravity. Therefore, the point of intersection of 3D force vectors in the horizontal plane can either:

**Remain stationary**. In this case the only possible changes in the fingertip force vectors are to increase or decrease their magnitudes simultaneously and proportionally, i.e., change the total grasp force. Mind the fact that these magnitudes are bounded above by finger strength and the possibility of crushing the object; and below by the need to support the object against gravity. Regardless of the location of the point of intersection within the object, such simultaneous and proportional increases or decreases in 3D fingertip force vector magnitudes will induce identically simultaneous and proportional changes in the normal component of the normal forces. This is captured by the Grasp Mode where all normal forces are positively correlated and therefore having PC loadings of the same sign, as in [0.81 0.41 0.41]^*T*^ in Figure 2. Please note that the loadings are the unit vectors describing the multidimensional correlation defining each PC. Therefore the loadings for this PC show that the thumb, index and middle finger forces all co-vary in this Mode. This analytical argument shows that the normal forces suffice to detect the spatial correlation structure defining the Grasp Mode. To confirm this, Figure 11 plots the loadings of the 1st PC of the 3D force analysis case (i.e., normal and two tangential forces for each digit) for all subjects and trials. This 9D equivalent to the Grasp Mode shows that positive correlation of all three normal forces dominates, and that the loadings of the tangential forces straddle the zero line (i.e., do not show strong covariation with the normal forces) to create a vector roughly [0 0 0.8 0 0 0.4 0 0 0.4]^*T*^.

**Move within the object**. If, say, the thumb force vector maintained its magnitude but changed its direction along an arc to the right by increasing its the tangential component and decreasing its normal component, then maintaining static equilibrium (as it was in the experiments we analyzed) would require the other two fingertip force vectors to track the 3D thumb force vector. In so doing, the magnitude of one fingertip force vector must increase, and the other decrease. This lengthening and shortening of the vectors must again be simultaneous and proportional. Once again, this will also induce identically simultaneous and proportional changes in the normal component of the normal forces. This is captured by the Compensation Mode, where one normal force is positively correlated with the thumb force and the other negatively. Thus the fingers have PC loadings of opposite signs, as in [0 −0.71 0.71]^*T*^ in Figure 2. That is, the full 3-component force vectors are not required to detect these changes. The normal forces suffice to detect these changes and their associated structure as the Compensation Mode. To confirm this, Figure 12 plots the loadings 2nd PC of the 3D force analysis case for all subjects and trials. This 9D equivalent to the Compensation Mode shows a dominant anti-correlation between the loadings of the normal forces of the index and middle finger, and that the loadings of the normal force of the thumb and all tangential forces straddle the zero line to create a vector roughly [0 0 0 0 0 −0.7 0 0 0.7]^*T*^.

A different combination of normal forces is the one perpendicular to the manifold. This is the “Hinge Mode” that would induce linear motion, with PC loadings [0.6 −0.5 −0.5]^*T*^ (thumb normal force increasing and simultaneous and proportional decreases in the fingers' normal forces). Our results show that dynamics along this task-relevant Mode was minimal because, by construction, we only analyzed cases where the object was in static equilibrium, in agreement with the UCM hypothesis that this Mode exhibits less variability. The normal forces suffice to detect these changes and their associated structure as the Hinge Mode, as also shown for the 3D force analysis case in Figure 13.

Very critically, we did not need PCA to identify our three Modes empirically. Rather, these were prescribed by the analytical solution to the mechanics of the system and task. PCA was only applied to the experimental data to identify for each subject the directions of normal force variability that maximally corresponded to the known directions inferred from mechanical analysis. For all subjects, these agreed well, by construction, with the closed-form analytical solution as mentioned in the results.

**Figure 7:**
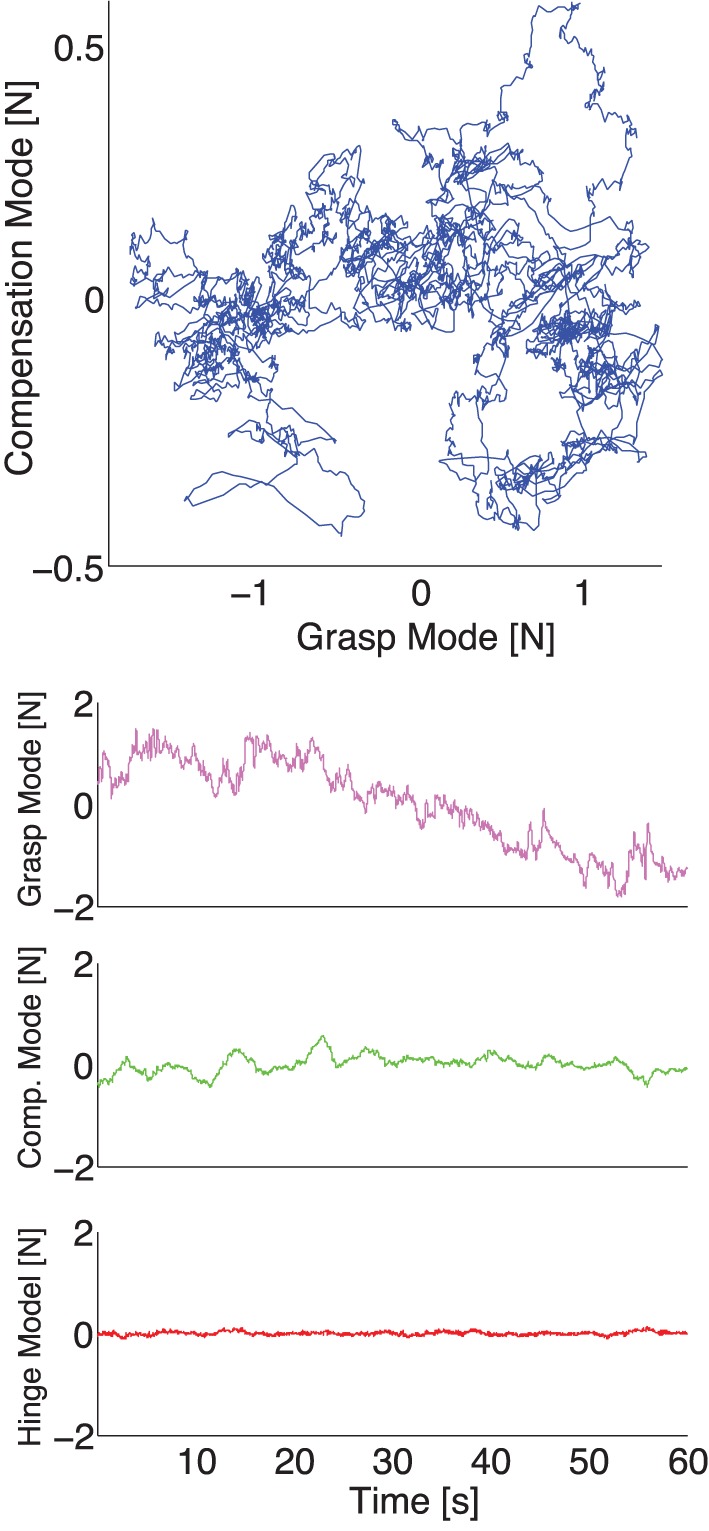
**Representative plot of the experimental normal forces now *projected onto the principal components* for the same representative trial as in Figure 4*without* visual feedback. Top:** The force fluctuations on the plane spanned by the Grasp and Compensation Modes. **Bottom:** The three principal component time series during a trial plotted individually. Note how the Grasp Mode captures the common downward trend, while the Compensation and Hinge Modes have relatively lower variability.

Our experimental spatial results, as expected, are in agreement with our simulations and the prior evidence for the UCM Hypothesis and the Minimal Intervention principle (Scholz and Schoener, 1999; Jordan, 2003): the variance in task-relevant variables is smaller than in the task-irrelevant spaces. The difference in variance explained by the Grasp and Hinge Modes in each case is explained by comparing Figures 4, 5 where, in the absence of visual feedback, the total grasp force (Grasp Mode) shows large variability that is absent when visual feedback is provided to avoid such drift. More specifically, the projection of the fingertip force time series data recorded without visual feedback onto the Grasp Mode shows a very slow monotonic downward trend (Figure 7 for a representative trial). We interpret this slow trend to be the major contributor to large spatial variability explained by this mode: it is caused by the three fingers reducing their normal forces simultaneously. This underscores an important shortcoming of PCA when applied to non-stationary signals (for a detailed discussion see Clewley et al., 2008). On the other hand, in trials *including* visual feedback, the Grasp Mode does not exhibit such a trend (Figure 5 for a representative trial). This is not surprising, since holding a constant total force is now an explicit task constraint, converting the Grasp Mode into a task-relevant Mode (see Table 3). As a consequence, the Compensation Mode (the other task-irrelevant dimension) now contributes a larger proportion of the overall variability (Figure 6). The fact that variability in the Grasp Mode does not disappear with visual feedback is well known and can be attributed to unavoidable motor noise, and other central and peripheral sources of correlated finger forces (Santello and Soechting, 2000; Poston et al., 2010; Rácz et al., (2012). The Compensation Mode also exhibits a slow non-monotonic modulation both increasing and decreasing over time (Figure 7). This indicates that index and middle finger normal forces are slowly and continuously modulated, out of phase, during static grasp.

**Table 3:** **Summary of findings, highlighting in bold discrepancy among UCM and Minimal Intervention (MI) predictions and temporal Detrended Fluctuation Analysis (DFA) results**.

**Mode**	**Experimental condition**	**Task relevance**	**UCM and MI predictions**	**Spatial PCA results**	**Temporal DFA results at different time scales**
Grasp mode	No visual feedback	Irrelevant	No control	High variance	Controlled and uncontrolled
	With visual feedback	Relevant	Control	Low variance	Controlled and uncontrolled
Compensation mode	No visual feedback	Irrelevant	No control	High variance	Controlled and uncontrolled
	With visual feedback	Irrelevant	No control	Low variance	Controlled and uncontrolled
Hinge mode	No visual feedback	Relevant	Control	High variance	Controlled and uncontrolled
	With visual feedback	Relevant	Control	Low variance	Controlled and uncontrolled

**Figure 8:**
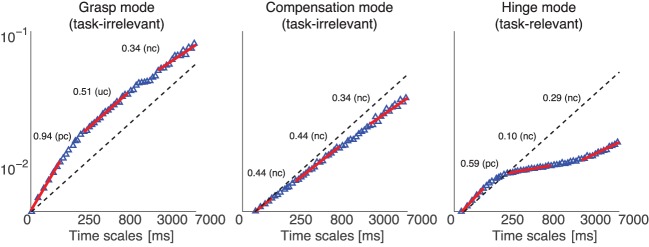
**Representative DFA of projected normal force time series from one subject, where the data were collected in a 200 g weight trial, *without* visual feedback**. The plot shows the three scaling regions (1–50, 250–500, and 3500–7000 ms) which we used to fit the scaling exponent, for each normal force correlation Mode (Grasp, Compensation, and Hinge Modes). The red lines show the linear fits to the behavior of diffusion vs. time scale—their slopes can either be greater than, equal or less than 0.5 (dashed line), indicating the diffusive process is positively correlated (pc or uncorrected divergence), uncorrelated (uc or Brownian motion), or negatively correlated (nc or corrective action), respectively, at those time scales.

**Figure 9:**
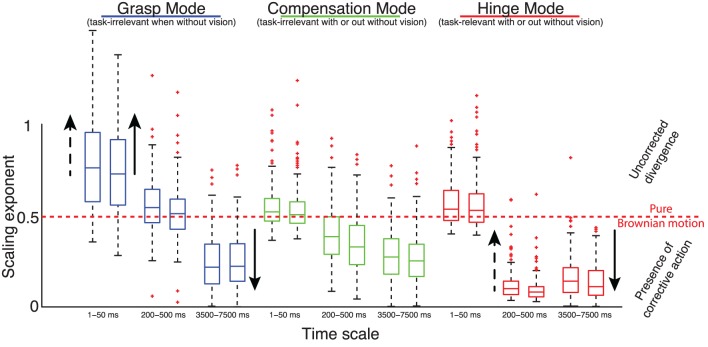
**Summary of temporal analysis**. Distribution of DFA scaling exponents of the normal forces projected onto the three Modes at three time scales—during the first (left box plots) and the second half (right box plots) of the trials, in trials *without* visual feedback. Solid arrows indicate the effect, if any, of adding visual feedback; and dashed arrows indicate the effect, if any, of increasing the weight. Note that these arrows indicate any statistically significant changes found based on the non-parametric statistical tests described in the text. We find that, contrary to the suppositions of the UCM hypothesis and Minimal Intervention principle borne by spatial analysis, at different time scales we see evidence of control effort (i.e., negatively correlated time histories with scaling exponents <0.5) in the task-irrelevant Modes (i.e., Grasp and Compensation); and evidence of uncorrected divergence in the Grasp Mode—which becomes task-relevant when visual feedback is provided—, and Brownian-like dynamics and unstable growth in the task-relevant Hinge Mode (i.e., non-correlated and positively correlated time histories, respectively, with scaling exponents ≥0.5).

As per Figures 8 and 9, by **uncontrolled** we mean evidence of unstable and Brownian growth (scaling exponents ≥0.5), and by **controlled** we mean evidence of corrective action (scaling exponents <0.5). In particular, both the Grasp and Compensation Modes are task-irrelevant but show temporal features of corrective action at some time scales. Similarly, the Hinge Mode is task-relevant but shows temporal features of lack of corrective action at some time scales.

As we have argued before (Clewley et al., 2008; Kutch and Valero-Cuevas, 2011, 2012), PCA of analytical solutions and experimental data alike naturally show a reduction in the dimensionality of task variables, which is a necessary result of meeting task constraints with a biomechanical plant. But this does not imply that the CNS is itself using a low-dimensional controller to simplify or optimize the redundancy problem. Rather, this simply reflects the structure of the solution space. Therefore, the question in not only whether the CNS *can* meet the requirements of the task (by definition it did if the task was accomplished), but also *how* it continues to meet them as time goes by. This makes temporal analysis of task variable dynamics critical to understanding the neural control actions in both the task-relevant and task-irrelevant spaces.

### Temporal analysis

Our DFA results, on the other hand, demonstrate the presence and absence of corrective actions by the CNS at different time scales in both the task-relevant and task-irrelevant task subspaces. Both linear and non-linear time series analysis has been commonly employed to reveal temporal correlation structures (positive or negative) indicative of control strategies (destabilizing or stabilizing, respectively), primarily in postural control research (Collins and De Luca, 1994; Jeka et al., 2004). For instance, in a seminal paper by Collins and De Luca (1994) the authors demonstrated a complex correlation structure in the center-of-pressure time series recorded during quiet stance, a highly redundant task. However, this perspective has not been brought to bear to the study of task redundancy. Once again, one can argue that the available literature endorses *preferential* as opposed to a strict separation into clearly controlled and uncontrolled variables, but we lacked a specific quantification of the temporal nature of the dynamics of task-relevant and task-irrelevant that would allow us to infer the neural control strategies in each space.

As per Figures 8, 9, we find that both task-relevant and task-irrelevant variables exhibit the features of uncorrected divergence, Brownian motion and corrective action, depending on the time scale considered—as evidenced by positive, neutral, and negative correlations between force increments separated by different time periods (i.e., scaling exponents >0.5, = 0.5, and <0.5, respectively). As per Table 3, the UCM and Minimal Intervention approaches would predict a clearer separation of corrective actions (i.e., control strategies) across task-relevant and task-irrelevant variables.

**Figure 10:**
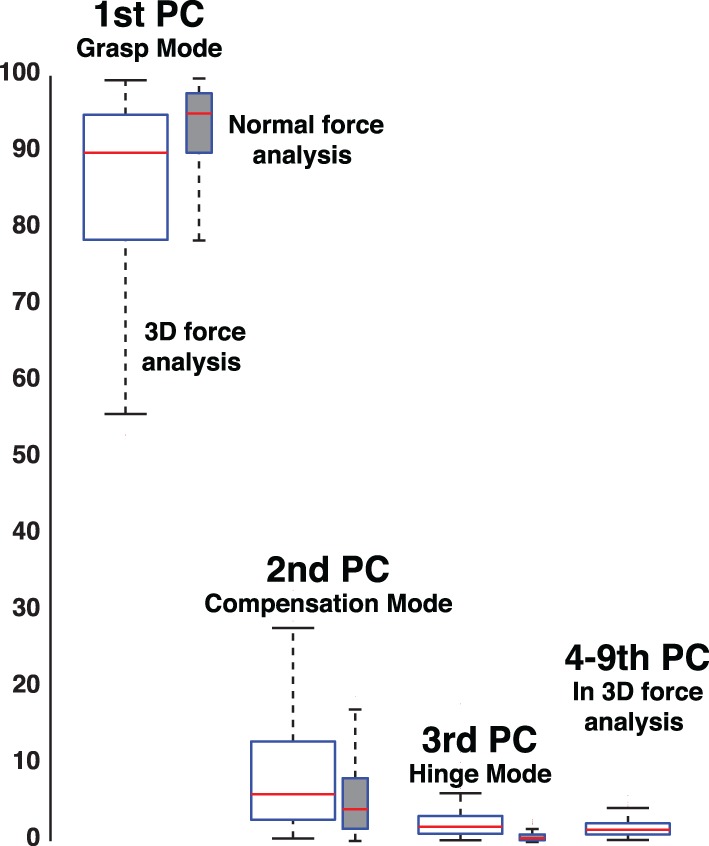
**Percent variance explained when considering three-dimensional forces for each digit (3D force analysis that is 9-dimensional given three forces for each of three digits, empty box plots) vs. when considering only the normal force at each digit (normal force analysis, gray box plots)**. The box plots show the variance explained by each PC from all subjects and trials, where the 1st PC explains the majority of the variance, the 2nd PC a modest amount, and the third PC less than 10%. The remaining variance explained by PCs four to nine is shown for the 3D force analysis. The structure of each PC is given by its loadings (as shown in Figures 11–13). Those figures shows that, even in the 3D force analysis case, the 1st, 2nd, and 3rd PC's represent the Grasp, Compensation and Hinge Modes seen in the normal force analysis. This consistency across percent variances explained demonstrates that the reduced normal force analysis is valid and equivalent to the full 3D force analysis.

**Figure 11:**
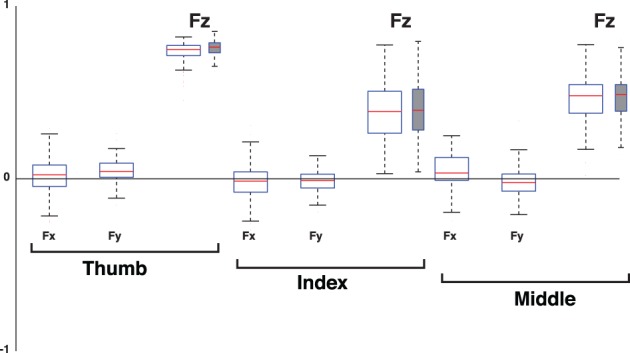
**Loadings of the 1st PC, the Grasp Mode, when considering the 3D force analysis case (normal plus two tangential force components, empty box plots) for each digit vs. when considering only its normal force (normal force analysis, gray box plots)**. Box plots show loadings from all subjects and trials. Note that the loadings of all tangential forces (Fx and Fy) straddle the zero line, demonstrating that they are not relevant to the correlation structure of the 1st PC. The normal force components (Fz) of all digits have positive and non-zero loadings, indicating that the structure of this PC using normal forces is equivalent to that of the full 3D force analysis. The dispersion or exact median values in the box plots are not the means to establish the task-relevance or task-irrelevance of the PC. That dispersion is a consequence of natural variability and inaccuracies in motor performance, and unavoidable sensor noise. It is the goals of the task and mechanical analysis that determine how to identify the task-relevant and task-irrelevant Modes.

**Figure 12:**
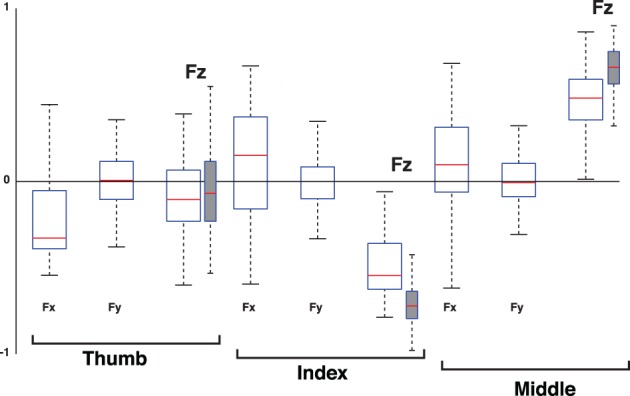
**Loadings of the 2nd PC, the Compensation Mode, when considering the 3D force analysis case for each digit (empty box plots) vs. when considering only its normal force (gray box plots)**. Box plots show loadings from all subjects and trials. Note that the loadings of the normal force components (Fz) of the thumb, and all tangential force components (Fx and Fy), straddle the zero line, demonstrating that they are not relevant to the correlation structure of the 2rd PC. The normal force components (Fz) of the index and middle finger exhibit anti-correlation, indicating that the structure of this PC using normal forces is equivalent to that of the full 3D force analysis. The increase in dispersion in the full 3D force analysis compared to the Grasp Mode in Figure 11 is naturally associated with the increased susceptibility to measurement noise as this Mode explains much less of the variance in the data, see Figure 10.

**Figure 13:**
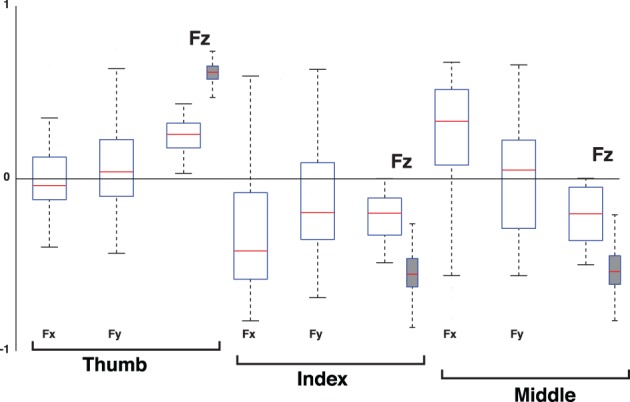
**Loadings of the 3rd PC, the Hinge Mode, when considering the 3D force analysis case for each digit (empty box plots) vs. when considering only its normal force (gray box plots)**. Box plots show loadings from all subjects and trials. Note that the loadings of the tangential forces (Fx and Fy) of the thumb, index and middle fingers straddle the zero line, demonstrating that they are not relevant to the correlation structure of the 3rd PC. The normal force components (Fz) of the thumb exhibits anti-correlation to those of the index and middle fingers, indicating that the structure of this PC using normal forces is equivalent to that of the full 3D force analysis. The increase in dispersion in the full 3D force analysis compared to the Grasp Mode in Figure 11 is naturally associated with the increased susceptibility to measurement noise as this Mode explains much less of the variance in the data, see Figure 10.

The temporal features of the task-irrelevant Grasp Mode challenge the UCM Hypothesis and the Minimal Intervention principle. The Grasp Mode (when no visual feedback is given) exhibits all three control strategies as the time scales lengthen, and goes from uncorrected divergence, to Brownian motion to corrective action. The slow downward trend in total grasp force in trials when without visual feedback happens at medium to long time scales—so it does not explain the uncorrected divergence seen at the short time scales. Such divergence, which was also present and even accentuated with visual feedback, is more likely a consequence of positive correlations that can be shown to be a result of the interplay between purely random signal-dependent noise (Jones et al., 2002), motor unit synchronization (Schieber and Santello, 2004), and instantaneous (but low-pass filtered by skin compliance) mechanical reaction forces. This variability in what are both task-irrelevant and task-relevant variables is nevertheless left uncorrected by the CNS either as part of the neural control strategy or because of inability to do so at such short latencies. Alternatively, we can argue that task-irrelevance is not only a spatial consideration but also a temporal one, where low-magnitude or short term variability is accepted and only corrected upon crossing a certain spatial or temporal threshold. But such interpretation is not really compatible with the UCM Hypothesis and the Minimal Intervention principle, but rather with other theories specifically phrased to advocate intermittent or drift-and-act control as an optimal strategy (Collins and De Luca, 1994; Guckenheimer, 1995; Milton et al., 2009a; Suzuki et al., 2012).

The neutral and negative correlations in the Grasp Mode at medium and long latencies, respectively, cannot be attributed to control intervention to avoid dropping the object due to a critical reduction in Grasp Mode force. The total grasp force level always remained well above the weight of the object, the hand was held still, and the scaling exponents were unchanged between the first and second half of the trials (Figure 9)—and slip-grip responses happen at latencies well below 200 ms (Cole and Abbs, 1988; Gysin et al., 2003; Rácz et al., 2012). Thus we conclude that corrective control intervention depends on factors other than safety boundaries or automatic grasp tendencies seen only during dynamic manipulation (Rácz et al., 2012). Moreover, such corrective control intervention occurs regardless of whether the Grasp Mode is task-irrelevant or task-relevant (when without or with visual feedback, respectively). Further challenging the UCM Hypothesis and the Minimal Intervention principle, the task-irrelevant Compensation Mode also exhibits corrective control intervention at medium and long time scales.

DFA exposes an absence of correlation at very short time scales in the task-relevant Hinge Mode. This indicates an absence of corrective actions (i.e., control). This lack of control may, however, simply be due to the inability of the neuromuscular system to do so at such short latencies; or may be evidence of an intermittent or drift-and-act strategy. While finding the reasons for this requires further investigation, it is nevertheless important to point out this important temporal feature not previously addressed by the UCM Hypothesis and the Minimal Intervention principle, to the best of our knowledge. That is, the fact remains that, due to physiological limitations or control strategy, even highly task-relevant variables are left uncontrolled at some time scales.

The fact that the results are so similar between the first and the second halves of the trials indicates that the observed dynamics and the associated correlation structure depend neither on time nor the total grasp force (which can be interpreted as location in the force space; or in control terms our findings are not state-dependent). This in turn suggests a temporal control strategy that is state-independent (except potentially *at* the boundaries; which we have no reason to believe our subjects approached, but could be an important next research step).

One possible explanation for the observed negative correlations along the Grasp and Compensation Modes could be that traversing the solution manifold is an active process, through which the CNS actually takes advantage of redundancy. Specifically, controlled dynamics along the Compensation Mode corresponds to the regulation of the index and middle finger contributions to the opposition of thumb normal force. In agreement with others, we speculate that the may be actively trying to shift the demands between the two fingers over time, which in turn might mitigate effects of fatigue at the muscle level (e.g., Cote et al., 2002; Dingwell et al., 2008; van Beers et al., 2013). By gradually varying fingertip forces, the CNS can achieve a change in the underlying muscle coordination pattern, which in turn will change the rates of fatiguing of individual muscles, thus allowing for improved use of available resources. The slow downward trend along the Grasp Mode direction of normal forces agrees with this fatigue reduction strategy: a general reduction of forces generated by the muscles leads to a reduction in the fatigue rate. But at these low levels of grasp force magnitude, the redundancy of solutions for a given set of fingertip force vectors would also allow changes in coordination patters that would not be detectable as changes in the magnitude or direction of fingertip force vectors. This issue, therefore, deserves further investigation.

Lastly, note that here we do not employ DFA to determine self-similarity or fractional dimensionality in the data, as has been done in some studies (Hausdorff et al., 1996). In those studies, the linearity in the logarithmic plots needs to extend over at least one order of magnitude to count as strong evidence of fractionality (Kantz and Schreiber, 2004). In our case the requirements for the linearity of the logarithmic plots are not as rigid because the quantification of long-range correlations applies to data where the linearity extends over shorter ranges of time scales. Moreover, challenging the preferential separation of control action across task variables as in the UCM Hypothesis and the Minimal Intervention principle only requires evidence of similar corrective actions (or their absence) in both task-relevant and task-irrelevant-which our results clearly show. These results expose a fundamental limitation of the UCM hypothesis and the Minimum Intervention Principle: their focus on spatial aspects of motor variability and disregard for temporal aspects.

### Conclusions and modularity

We show that both task-relevant and task-irrelevant parameters show corrective action at some time scales; and conversely, that task-relevant parameters do not show corrective action at other time scales. In agreement with recent work in other behavioral contexts, these results propose we revise our understanding of variability vis-á-vis task relevance by considering both spatial and temporal features of all task variables when inferring control action and understanding how the CNS confronts task redundancy. Moreover, these results are counter examples to the UCM hypothesis and the Minimal Intervention principle, as they assume a separation of task variables into relevant and irrelevant ones, indicated by their respective variabilities. As mentioned above, proponents of UCM hypothesis and the Minimal Intervention principle admit the possibility of *preferential* as opposed to strictly *uncontrolled* variables (Latash et al., 2010), or that the nervous system largely “ignores non-essential variations” (Dingwell et al., 2010), but such qualitative distinctions have only begun to be quantified or considered in the spatio-temporal domain when inferring control action. Following up on those qualifications, we present specific spatio-temporal quantitative examples of controlled intervention (or lack thereof) in both task-relevant and task-irrelevant spaces (based on mechanical/mathematical definition of the task and its possible modes of variability) to expand our understanding of neural control strategies. Additional work is needed to revise our view of neural control that takes into considerations both spatial and temporal aspects of neuromuscular function and variability, and the structure and nature of the solution space of the task.

The similar nature of control actions across time scales in both task-relevant and task-irrelevant spaces that we find point to a level of modularity not previously recognized. The spatio-temporal results presented here instead suggest that neural control uses a continuum of control strategies going from uncorrected divergence to strong corrective actions that are not defined by the level of task-relevance of the controlled variables; and which may also involve intermittent and drift-and-act characteristics. Importantly, while the increase in weight and the addition of visual feedback does seem to modulate the dynamics on the individual dimensions, it does not lead to a crossing of the 0.5 line and therefore not to a fundamental change in the control strategy. Our methodological consideration and spatio-temporal analysis allow us to present clear examples of how the task-irrelevant parameters (i.e., elemental variables that are organized to constitute the UCM) are actively and continuously controlled during a tripod grasp at certain time scales, while the task-relevant parameter (or performance variable) is not actively controlled during a tripod grasp at certain time scales. Therefore, we show that estimating the different extents of control based on task variable variances alone (a purely spatial approach) is insufficient, as Dingwell and we had proposed before (Valero-Cuevas et al., 2009; Dingwell et al., 2010). Rather, those variables constituting the UCM (which are again, mathematically defined by the unambiguous mechanics of the task, see Figure 2) may have different temporal dynamics, but are not controlled in a fundamentally different way.

This spatio-temporal approach to variability provides a tool to quantify the nature and degree of neural control action, extending the traditional spatial variance magnitude approach by quantifying the temporal nature of variability. For example, traversing the solution manifold is an active process by which the controller *enforces* the constraints of the task. The CNS does not *create* the solution manifold^1^, but rather seeks to inhabit it as has been discussed earlier (Keenan et al., 2009; Kutch and Valero-Cuevas, 2012; Suzuki et al., 2012). As such, the means by which the CNS enters and continually inhabits the solution manifold can be thought of as the implementation of a dynamical attractor on the task variables. In the context of time-varying stochastic behavior of differential and discrete-time distributed systems like the neuromuscular system, the implementation of such a controller enforcing an attractor can be thought of as the implementation of a specific probability density of the state (for a presentation of this view see Sanger (2011), which is different from Bayesian estimation and discrete-time Markov processes).

This emerging view of the nervous system as functioning at the level of affecting probability density functions (Sanger, 2011) is compatible with a modular interpretation of our spatio-temporal results. DFA estimates the statistical self-affinity of stochastic processes with memory whose underlying statistics (mean, standard deviation and higher-order moments) or dynamics are non-stationary (Kantelhardt et al., 2001). That is, DFA quantifies how well a probability density function is implemented. Thus the continuum of control strategies seen across all Modes and time scales can be thought of as essentially differently tuned versions of the same modular control process that can let drift (i.e., uncorrected divergence), be indifferent, or enforce (i.e., corrective action) the statistics of the time-varying probability density of the state so that it populates the solution space. Hence the level of modularity in the controller rests on the ability of the system to work with probability density functions in the task-relevant and task-irrelevant spaces at different time scales—and not with distinct basis functions or synergies implementing a separation of task variables.

### Conflict of interest statement

The authors declare that the research was conducted in the absence of any commercial or financial relationships that could be construed as a potential conflict of interest.

## Footnotes

^1^To be clear, the solution manifold arises independently of the controller as it depends only on the characteristics of the plant and the constraints of the tasks. A controller can then choose to inhabit a particular region or subset of the solution manifold to meet the requirements of the task (Kutch and Valero-Cuevas, 2012; Suzuki et al., 2012).

## Appendix

### Identification and modeling of the mechanical requirements of the task and its null space

Each fingertip applies a three-dimensional force $\tilde{f}$ to the object. Computing the cross product of the moment arm, i.e., the vector between the point of force application and the object's center of mass, with the fingertip force vector yields the moment applied to the object. The total 6-dimensional force and moment applied to the object can be computed with the following mapping **W**:

$$\begin{array}{l} {\left[\begin{array}{c} \sum f\\ \sum m \end{array}\right]}_{6\times 1}={\left[\begin{array}{lll} I_{3\times 3} & I_{3\times 3} & I_{3\times 3}\\ M_{\text{th}} & M_{\text{ind}} & M_{\text{mid}} \end{array}\right]}_{6\times 9}{\left[\begin{array}{c} {\tilde{f}}_{\text{th}}\\ {\tilde{f}}_{\text{ind}}\\ {\tilde{f}}_{\text{mid}} \end{array}\right]}_{{}_{9\times 1}}\\ \;=W\tilde{f} \end{array}$$

where **I**_3 × 3_ is the unit matrix and **M**_{th, ind, mid}_ is the skew-symmetric matrix representing the cross-product between the moment arm of the finger and its force vector **f**_{th, ind, mid}_. Since **W** is a mapping from 9-dimensional (three 3-D finger forces) to 6-dimensional (six degrees of freedom for the object grasped) space, the associated null space, i.e., the space of vectors for which $\tilde{0}=\text{W}\tilde{x}$ has 3 dimensions. Any vector $\tilde{x}$ in this null space represents a solution to the static grasp requirement $\left[\begin{array}{c} \sum f\\ \sum m \end{array}\right]=\left[\begin{array}{c} \tilde{0}\\ \tilde{0} \end{array}\right]$, i.e., that both the sum of forces and the sum of moments should be zero. This is the mathematical description of the task-irrelevant subspace because fingertip forces can change inside this space but the object will remain static.

However, this is a necessary, but not sufficient, requirement. Additionally, we require that the finger tips do not slip, so the tangential forces are upper-bounded through the friction relationship *f*_tangential_ ≤ μ*f*_normal_, i.e., the tangential force cannot exceed the normal force, multiplied with the friction coefficient μ, which we have set to 0.04, the approximate friction coefficient of Teflon on Teflon. This represents a lower bound on the coefficient of friction, since this coefficient is certainly greater when fingertip and Teflon surface interact. That is, the grasp under experimental conditions is actually less constrained and tangential components can be greater. In addition, the sum of tangential forces directed vertically needs to oppose the force applied to the object by gravity. A nominal object had a weight of 100 g, hence the sum of tangential forces had to equal 0.981N, which in turn determined the sign (positive, i.e., into the object) and the minimum magnitude of the normal forces. This requirement changes with the weight condition, of course. For a complete list of static grasp model constraints, see Table A1.

**Table A1:** **List of relevant constraints in static grasp**.

**Constraint**	**Magnitude**	**Interpretation**
∑ **F**	[0, 0, *mg*]^*T*^	Sum of forces equal and opposite to gravity force, i.e., no net force
∑ **M**	**0**	Sum of moments equals zero, i.e., no net moment
*F*_*i*__*T*_	≤μ · *F*_*i*__*N*_	Tangential force at the *i*-th finger cannot exceed normal force

Given that the task null space is well described, it was important to simulate the properties of expected solutions to compare against the experimental results to properly disambiguate the spatio-temporal features of the fingertip forces that can be explained by mechanics from those of neural origin; as in Rácz et al. (2012) and Kutch and Valero-Cuevas (2012). Enforcing all constraints gives us mathematical description of the null space of the task. To simulate instances of these fingertip forces, we numerically sampled vectors ${\tilde{f}}_{t_{\text{null}}}$ from the null space of the above linear matrix by multiplying the three null space basis vectors ñ_*i*_ with random values *a, b, c*, drawn from a standard Brownian random walk: ${\tilde{f}}_{t_{\text{null}}}=a\cdot {\tilde{n}}_{1}+b\cdot {\tilde{n}}_{2}+c\cdot {\tilde{n}}_{3}$. We then added these null space vectors ${\tilde{f}}_{\text{null}}$ to the minimum sum-of-squared-forces solution ${\tilde{f}}_{\text{min sq}}$ of force vectors that met all the above described static grasp constraints: ${\tilde{f}}_{t}={\tilde{f}}_{\text{min sq}}+{\tilde{f}}_{t_{\text{null}}}$, using MATLAB's (Natick, MA) quadprog() function to determine the actual solution with minimum Euclidean distance to ${\tilde{f}}_{\text{min sq}}+{\tilde{f}}_{t_{\text{null}}}$.
